# T229. ANTIPSYCHOTIC DRUG USE AND THYROID FUNCTION IN PATIENTS WITH SEVERE MENTAL DISORDERS

**DOI:** 10.1093/schbul/sby016.505

**Published:** 2018-04-01

**Authors:** Trude Iversen, Nils E Steen, Kåre I Birkeland, Ragni H Mørch, Elina J Reponen, Jannicke F Anderssen, Linn Rødevand, Ingrid Melle, Ole A Andreassen, Espen Molden, Erik Jönsson

**Affiliations:** 1 University of Oslo, Oslo University Hospital; 2 Diakonhjemmet Hospital; 3 Oslo University Hospital, University of Oslo, Karolinska Institutet

## Abstract

**Background:**

Altered levels of free Thyroxin (fT4) and Thyroid Stimulating Hormone (TSH) have been associated with severe mental disorders and the use of antipsychotic drugs. Still, there is a lack of studies systematically investigating commonly prescribed antipsychotic drugs and thyroid function. We investigated the association of antipsychotic drugs and thyroid hormones levels in patients with severe mental disorders and compared thyroid function tests between patients and healthy controls under real-life conditions.

**Methods:**

We included 1345 patients with schizophrenia or bipolar disorders and 989 healthy controls from the on-going Thematically Organized Psychosis (TOP) study, recruiting participants between 18–65 years of age in and around Oslo, Norway. All patients underwent a thorough clinical investigation including diagnostic evaluation, somatic screening and assessment of medication data. Serum drug concentrations were measured. Plasma levels of fT4 and TSH were measured in patients and healthy controls, and thyroid status was determined based on the combined hormone levels. Participants with known thyroid function disorders (N=28) were excluded. Mann-Whitney U tests and chi-square tests were performed for comparison between groups. For evaluation of influence from antipsychotics, multiple linear regression analyses were performed, adjusting for patient/control status, age, sex and use of other psychopharmacological agents. Associations with the use of olanzapine, quetiapine, aripiprazole or risperidone in monotherapy were analyzed in a subsample of patients (N=480), adjusting for age, sex and diagnosis. Spearman correlation analyses were performed for hormone levels and drug serum concentrations.

**Results:**

We found significant lower levels of fT4 (median 13.70 vs 14.00, p<0.001) and higher levels of TSH (median 1.92 vs 1.57, p<0.001) in patients compared to healthy controls. A significant difference between patients and controls in occurrence of hyper- and hypothyreosis was observed (p<0.001), with more than three times as many patients compared to controls with hypothyroid status (11.1% vs 3.4%), and a doubling of hyperthyroid status (2.3% vs. 1.2%). Use of antipsychotics was significantly associated with lower fT4 level (p=0.001), but not with the TSH level. We also found significant associations between lower fT4 level and current use of quetiapine (p=0.005) and olanzapine (p=0.018), but again no significant associations were found with TSH level. No significant correlations were found between drug serum concentrations and fT4 or TSH. In the regression analyses we also observed that female sex and increasing age was associated with lower levels of both fT4 and TSH.

**Discussion:**

In this large, cross-sectional study we found significant differences in thyroid hormone levels between patients with schizophrenia and bipolar disorders and healthy controls, and our data indicate a notable prevalence of undetected deviant thyroid function in the patient population. There was a significant association between fT4 level and the use of antipsychotics, particularly with the use of quetiapine and olanzapine. This suggests a possible contribution to altered thyroid hormone levels from the use of commonly prescribed antipsychotic agents. These findings call for renewed attention towards the role of thyroid function in severe mental disorders and the associations with antipsychotic drugs.

